# By the Way

**Published:** 1912-11-23

**Authors:** 


### BY THE WAY.

### FIFTY YEARS AGO.

A COMPLIMENTARY dinner was once given in Buffalo to two doctors of that city in celebration of the fiftieth anniversary of their graduation. Each of the veterans, in responding to the compliments of their friends after the feast, read a paper full of reminiscences of older days, and one of their patients, Judge Hurd, spoke feelingly on the experiences of a patient fifty years ago. Finally Dr. Booth contributed a poem, from which we quote the concluding stanza:—

Ship me somewhere back of sixty
  Where the best was like the worst,
And the man who drove the hardest
  Got to treat the case the first;

When the dear old code of ethics
  With its fine distinctions clear
Was simply used to guide the few
  Who held traditions dear.

On the road to yesterday, where our masters led
  the way,
With their purging blue-mass doses, and their lancet
  to the fray.
On the road to yesterday, where we fellows fain
  would stay
For our minds are stunned, by thunder! at the
  change there is to-day.

### UNRELIABLE PATIENTS: A FRENCH VIEW.

THE unreliable patient is of two kinds. Either he is discontented with his medical adviser or else, on the contrary, after a single consultation he sings the latter's praises beyond measure. The universe is not sufficiently vast to contain with befitting grandeur the words of admiration which he pronounces in honour of his saviour. Flattered, the doctor allows things to take their course. After all, what possible object could he have in protesting? Still he ought to be cautious, and to fight shy of this pyrotechnic display of adulatory language. Once the rocket has gone off there remains but a wretched cylinder of cardboard difficult to set alight. Spread abroad at random, the eulogies quickly take on a less warm tone. The patient now takes stock of his attendant to make sure that he has not been duped. He has not found on all sides the echo to his own enthusiasm. Some have replied disparagingly, and he now begins to take heed of their remarks. Truth to tell, perhaps, he has been a little too fulsome in his praises? His critical faculty returns after a preliminary explosion, and it is a somewhat lukewarm esteem that now animates him. Then must the doctor take precautions. His examinations must be complete, minute, and detailed, for the patient is determined not to be taken in a second time. He is for ever on the watch, spying, his mind made up to refuse his confidence and to transfer it elsewhere on the least indication of distraction or weakness on his former hero's part. There is no one so hard to retain as the patient who too quickly and too warmly has cried out one's merits from the housetops. Again, there are moments when the physician is off his guard. He has confidence in his patient and examines him with less than his usual care, talks, and is distracted. The person interested does not pardon this. Once outside he quickly establishes the truth. This man a doctor? A charlatan, negligent, a talker, distracted and ignorant. You have here an *embarras du choix*. It is well to be aware of this mentality of a certain type of patient. It is to be seen in your new cases, in those whom your first visit has upset with hope or despair. Every strong emotion has a tendency to die out, and it is the opposite sentiment which on most occasions arises from its ashes. If nothing is so akin to love as hate, nothing so nearly approaches an exaggerated admiration as an absolute contempt. No doubt a certain number of patients remain faithful. They are those who, having warmed up less quickly, are not in need of recovering their equilibrium by throwing themselves into the extremes of the opposite sentiment. In the presence of explosive natures the physician has but one way of preserving their confidence: he must be in no hurry, must listen patiently, and must talk to them in an affirmative manner tempered by an accent of kindliness. At this price the constancy of a patient may be acquired, and once a cure has been wrought a sincere and abiding gratitude may place him among those faithful to you.—*Journal des Praticiens.*

